# Rapid and accurate quantification of isomiRs by RT-qPCR

**DOI:** 10.1038/s41598-022-22298-7

**Published:** 2022-10-14

**Authors:** Sandra Franco, Raquel Pluvinet, Jose Francisco Sanchez-Herrero, Lauro Sumoy, Miguel Angel Martinez

**Affiliations:** 1 IrsiCaixa, Hospital Universitari Germans Trias i Pujol, Universitat Autònoma de Barcelona (UAB), 08916 Badalona, Spain; 2 Institut Germans Trias i Pujol (IGTP), Badalona, Spain

**Keywords:** Biological techniques, Microbiology, Molecular biology

## Abstract

Currently, microRNAs (miRs) are annotated as a single defined sequence (canonical), even though high-throughput small RNA sequencing has identified miR isoforms (isomiRs) that differ from their canonical counterparts in length, sequence, or both. Here we describe a simple reverse transcriptase-quantitative polymerase chain reaction (RT-qPCR)-based assay for quantification of the miR-100-5p_iso_3p:−2 variant. We chose miR-100-5p because the canonical sequence was underrepresented in our evaluation of human plasma. The quantification of miR-100-5p_iso_3 p:−2 from 57 plasma samples demonstrated high concordance between high-throughput RNA sequencing and RT-qPCR results (*r* = 0.55, *p* < 0.0001). Of note, we could not detect or quantify miR-100-5p in our plasma samples using a commercial TaqMan canonical miR-100-5p RT-qPCR kit. With these 57 samples, we also adapted this assay to specifically quantify the canonical sequences of miR-122-5p and miR-192-5p. Similar to the results obtained with miR-100-5p_iso_3p:−2, RT-qPCR results for miR-122-5p and miR-192-5p highly correlated with high-throughput RNA sequencing data (miR-122-5p: *r* = 0.44, *p* = 0.0005; miR-192-5p: *r* = 0.72, *p* < 0.0001). The assay described here can be easily adapted to many different identified isomiRs. Because of the high specificity of isomiRs, their reliable RT-qPCR-based quantification could provide greater resolution and higher accuracy than using canonical sequences.

## Introduction

MicroRNAs (miRs) are single-stranded noncoding RNAs consisting of 19–22 nucleotides. In animals, miRs act as post-transcriptional inhibitors of gene expression. They are implicated in almost every cellular process and are critical for cell differentiation, homeostasis, and animal development^1^. Computational analyses indicate that more than 50% of the human transcriptome may be regulated by at least one miR^2^. Soon after the discovery of miRs 30 years ago, changes in cellular miR levels were found to be related to specific cell or tissue pathology. These findings opened up new opportunities for disease diagnosis. Indeed, abnormal expression of miRs is associated with multiple diseases including cancers, viral infections, liver injury, diabetes, and cardiovascular and neurodegenerative diseases. Of interest, miRs are secreted into extracellular fluids and delivered to other cells where they also can function^3^, suggesting a possible role for miRs as signalling and coordination molecules between cells and organs. These findings have highlighted miRs as excellent disease biomarkers^4,5^.

miRs are easily accessible in body fluids, such as blood, urine, or saliva, using noninvasive methods and are stable and readily measurable by several techniques. These methods mainly include sequencing of small RNA, reverse transcriptase-quantitative polymerase chain reaction (RT-qPCR), microarray analysis, and northern blot. RT-qPCR is a reliable, sensitive, simple, fast, and cost-effective approach to quantifying circulating miRs^6^, which has led to widely used commercial RT-qPCR–based assays for miR detection and quantification (e.g., Exiqon’s LNA miRCuRY qPCR and Applied Biosystems’s TaqMan MicroRNA qPCR). Although these miR qPCR assays show high sensitivity for specific sequences, they also detect background signals from closely related miR isoform (isomiR) sequences, which may affect miR quantification^7,8^. Moreover, these commercial assays, designed to detect the canonical miR sequences, can be problematic for detecting physiologically relevant isomiRs.

High-throughput sequencing of small RNAs has revealed that miRs frequently appear in the form of multiple sequence variants or isomiRs^9^. IsomiRs can differ from their respective reference (i.e., canonical) miRs by one or more nucleotides. Variations can occur at the 5′ or 3′ ends of the miR where bases can be added or subtracted, or anywhere within the sequence. These isomiRs may originate through imprecise or alternative cleavage during pre-miR processing and by post-transcriptional modifications^10^. IsomiRs can have not only different target specificity but also different stability and/or different subcellular localisation^11,12^. Recently, single-cell small RNA sequencing has revealed altered regulatory functions of different classes of isomiRs when compared with their respective canonical miRs, supporting a biological role for many of the expressed isomiRs^13^. We and others have demonstrated that isomiRs can be more informative biomarkers than their corresponding canonical miRs^14,15^.

As noted above, commercial RT-qPCR assays targeting miRs designed to recognise the canonical forms can show variable degrees of specificity for the different corresponding isomiRs. Moreover, we have recently demonstrated that even small RNA sequencing can lead to overestimation of the abundance and diversity of isomiRs as a consequence of library preparation biases, sequencing and/or data processing errors^16,17^. Here we show how a previously described fast, simple, and cost-effective RT-qPCR-based assay^18^ can be adapted for detection and precise quantification of either isomiR or canonical miR sequences. This assay can be easily adjusted for different identified isomiRs to improve the statistical capacity of differential expression analyses and overcome the associated problems observed in some instances with commercial RT-qPCR assays.

## Results

**Figure 1:**
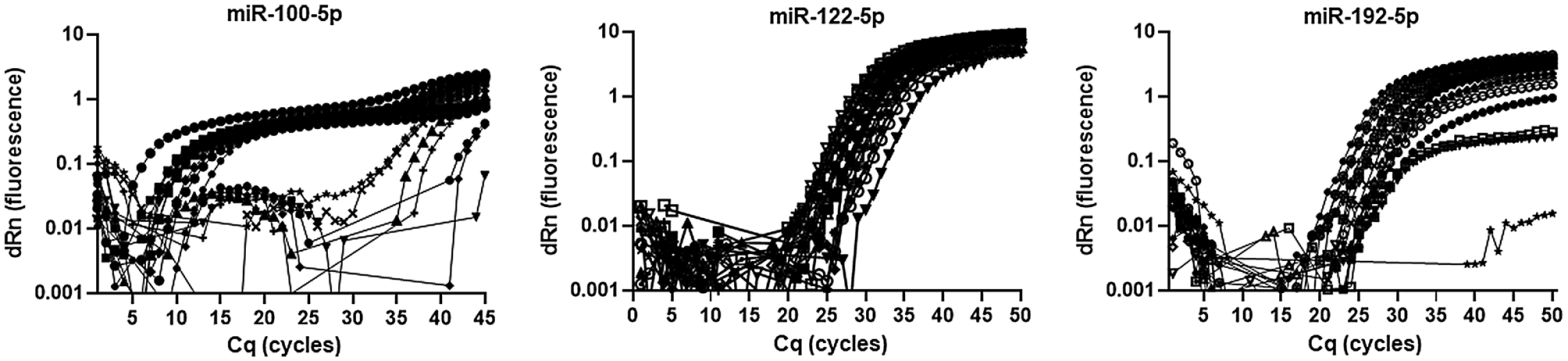
RT-qPCR TaqMan assay measurement of miR-100-5p, miR-122-5p, and miR-192-5p. Amplification TaqMan plots of representative plasma samples. In contrast to miR-122-5p and miR-192-5p, miR-100-5p was not detected by the commercial TaqMan assay. Measurements were set up in duplicate.

In a previous study, we performed a large-scale deep sequencing analysis of small RNA expression in plasma samples from individuals coinfected with human immunodeficiency virus type 1 and hepatitis C virus (HIV-1/HCV) and uninfected healthy controls^15^. Among an average of 3.2 million miR reads per sample, we detected 1065 different miRs across all analysed plasma samples. In this study, we identified a signature of three miRs (miR-100-5p, miR-122-5p, and miR-192-5p) that was highly correlated with progression to liver fibrosis^15^. To confirm the clinical validity of our miR signature and validate its predictive performance in an independent prospective validation cohort by RT-qPCR–based miR detection, we used the commercially available TaqMan miR assay. With this assay, we quantified the plasma expression of the previously identified miR signature in 57 plasma samples analysed by small RNA sequencing^15^. With this commercial assay, we were unable to detect and quantify miR-100-5p (Fig. 1). In contrast, miR-122-5p and miR-192-5p were detected and appropriately quantified.

The small RNA sequencing data analysis of our 57 previously studied plasma samples from individuals with and without HIV-1/HCV coinfection showed a low expression of the canonical miR-100-5p variant (Table 1). A miR-100-5p iso_3p:−2 variant was the most abundant sequence followed by an iso_3p:−1. This finding suggests that the lack of the canonical miR-100-5p sequence in our samples could explain why the commercial TaqMan assay failed to detect this miR. In contrast to miR-100-5p, the canonical variants of miR-122-p and miR-192-5p were highly expressed (Table 1), again strongly suggesting why the commercial TaqMan assay could detect and accurately quantify these miRs.

**Table 1:** miR variant expression as determined by high-throughput small RNA sequencing.

miR	isomiR variant	BaseMean^a^	Sequence
hsa-miR-100-5*p*	iso_3p:−2	675.07	AACCCGUAGAUCCGAACUUG
	iso_3p:−1	280.31	AACCCGUAGAUCCGAACUUGU
	iso_3p:−4	18.71	AACCCGUAGAUCCGAACU
	Canonical	15.68	AACCCGUAGAUCCGAACUUGUG
	iso_3p:−3	7.85	AACCCGUAGAUCCGAACUU
hsa-miR-122-5*p*	iso_3p:−1	60.32	UGGAGUGUGACAAUGGUGUUU
	Canonical	48.53	UGGAGUGUGACAAUGGUGUUUG
	iso_5p:−1	20.37	GGAGUGUGACAAUGGUGUUUG
	iso_5p:−2	18.10	GAGUGUGACAAUGGUGUUUG
	iso_3p:−2	8.98	UGGAGUGUGACAAUGGUGUU
hsa-miR-192-5*p*	Canonical	32,708.45	CUGACCUAUGAAUUGACAGCC
	iso_5p:−1	6749.04	UGACCUAUGAAUUGACAGCC
	iso_3p:−1	2599.87	CUGACCUAUGAAUUGACAGC
	iso_add: + 1	242.82	UGACCUAUGAAUUGACAGCCAGA
	iso_5p:−1	238.73	UUACCUAUGAAUUGACAGCC

^a^The average of the normalised count values according to DeSeq2 as described in (15).

**Figure 2:**
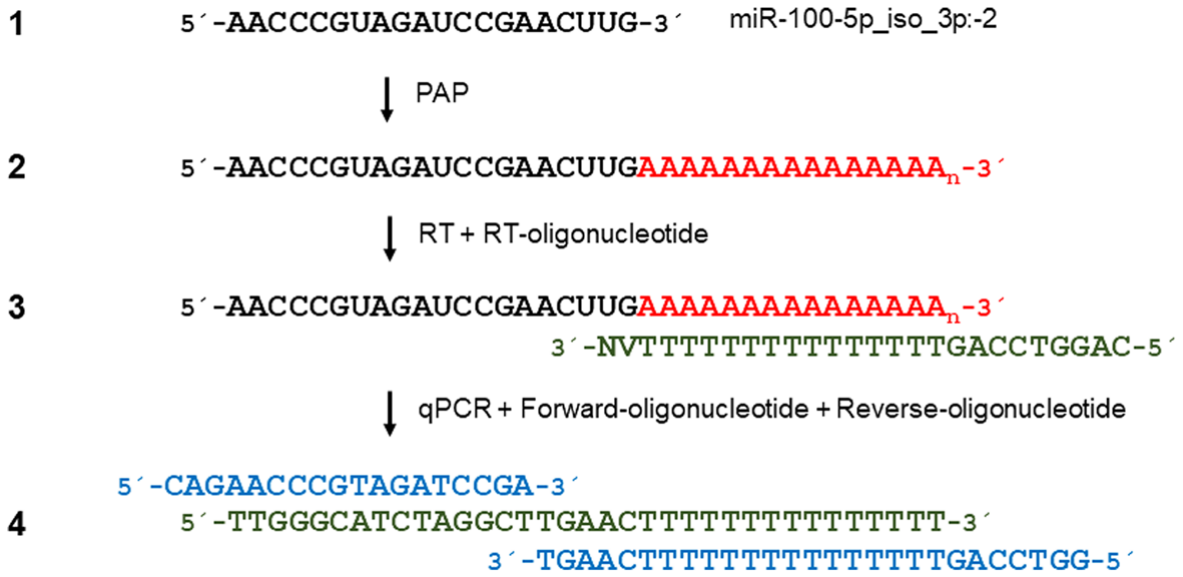
Schematic description of miR-specific RT-qPCR for miR-100-5p_iso_3p:−2 detection. This method uses SYBR Green detection chemistry and a single reverse transcription reaction for all miRs combined with real-time PCR with two miR-specific DNA oligonucleotides. Purified RNA containing miRs (1) is polyadenylated with adenosine and poly adenosine polymerase (PAP) (2), polyadenylated RNA is reverse transcribed with RT and an anchored poly deoxythymidine oligonucleotide (3), and the generated cDNA is then subjected to qPCR with two tagged, specific oligonucleotides (miR-100-5p_iso_3p:−2) (4).

**Figure 3:**
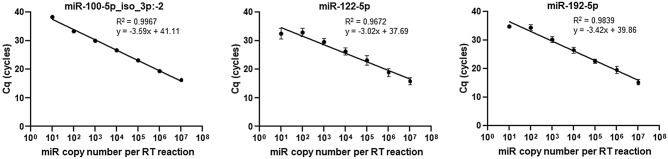
Dynamic range and sensitivity of the evaluated RT-qPCR. Standard curves for miR-100-5p_iso_3p:−2, miR-122-5p, and miR-192-5p assayed with the study RT-qPCR assay. As an internal positive control, 10^4^ copies of the cDNA derived from each miR synthetic oligonucleotide were added to confirm the detection onto the standard curve. As negative controls, we tested 10^4^ copies of the other miRs and RNU6B to ensure no cross-amplification. The RT-qPCR assay exhibited an excellent linearity between the log of the miR input and Cq values over seven orders of magnitude and accurately quantified down to 10 cDNA copies of miR. Measurements were set up in quintuplicate (Supplementary Table 1). Pearson correlations were used for statistical analysis.

To specifically and cost-effectively quantify expression of miR-100-5p, we adapted a previously described RT-qPCR method for miR quantification^18^. This method uses SYBR Green detection chemistry and a single reverse transcription reaction for all miRs combined with real-time PCR with two miR-specific DNA oligonucleotides (Fig. 2). In this method, purified RNA containing miRs is polyadenylated with adenosine and poly adenosine polymerase. Next, polyadenylated RNA is reverse transcribed with RT and an anchored poly deoxythymidine oligonucleotide. The resulting cDNA is subjected to qPCR with two tagged specific oligonucleotides. We designed specific oligonucleotides for the amplification and quantification of the miR-100-5p_iso_3p:−2 variant (Fig. 2) and the canonical miR-122-5p and miR-192-5p sequences. To confirm the specificity of the described assay, we applied melting curve analysis (Supplementary Fig. 1). The sensitivity and dynamic range of the described RT-qPCR were evaluated using synthetic miR-100-5p, miR-122-5p, and miR-192-5p as targets. A cDNA dilution series spanning eight orders of magnitude was prepared ranging from 10 to 10^7^ copies of miR-100-5p_iso_3p:−2, miR-122-5p, or miR-192-5p molecules per RT reaction. The RT-qPCR assay exhibited an excellent linearity between the log of the miR input and quantification cycle (Cq) values over seven orders of magnitude and accurately quantified down to 10 cDNA copies of miR-100-5p_iso_3p:−2, miR-122-5p, or miR-192-5p corresponding to 10 miRs in the original sample (R^2^ = 0.9967, R^2^ = 0.9672, and R^2^ = 0.9839, respectively; Fig. 3, Supplementary Table 1). The intra-assay coefficients of variation for the three tested miRs were 0.81% for miR-100-5p_iso_3p:−2, 0.97% for miR-122-5p, and 1.60% for miR-192-5p. The inter-assay coefficients of variation with these three miRs also were good (0.96%, 2.55%, and 1.72%, respectively). Of note, canonical miR-100-5p designed oligonucleotides were not able to amplify a synthetic miR-100-5p_iso_3p:−2 cDNA target template and shorter isomir cDNA target templates, but did amplify synthetic miR-100-5p_iso_3p:−1 at 24% cross-reactivity (Supplementary Fig. 2).

**Figure 4:**
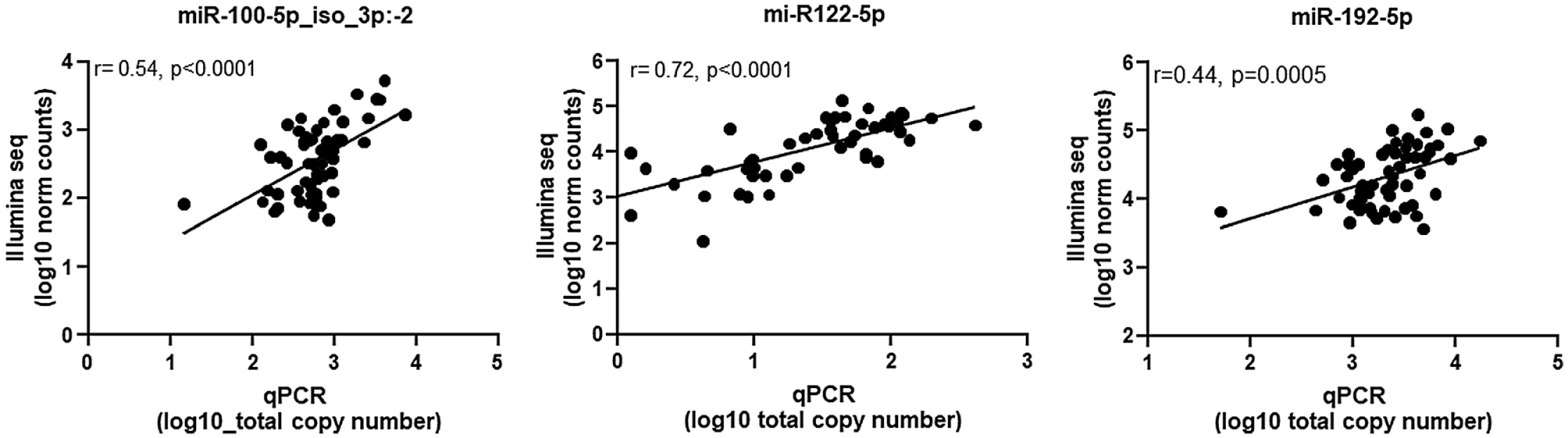
Correlation of plasma miR-100-5p_iso_3p:−2, miR-122-5p, and miR-192-5p levels determined by RT-qPCR and Illumina large-scale deep sequencing analysis. Sequence reads of the miR-100-5p_iso_3p:−2 variant and those from the canonical miR-122-5p and miR-192-5p quantified by large-scale deep sequencing on 57 plasma samples obtained from individuals coinfected with HIV-1/HCV and uninfected healthy controls, correlated with the study RT-qPCR assay results. Correlation between the two datasets was good for each of the three miRs analysed. Pearson correlation was used for statistical analyses.

**Figure 5:**
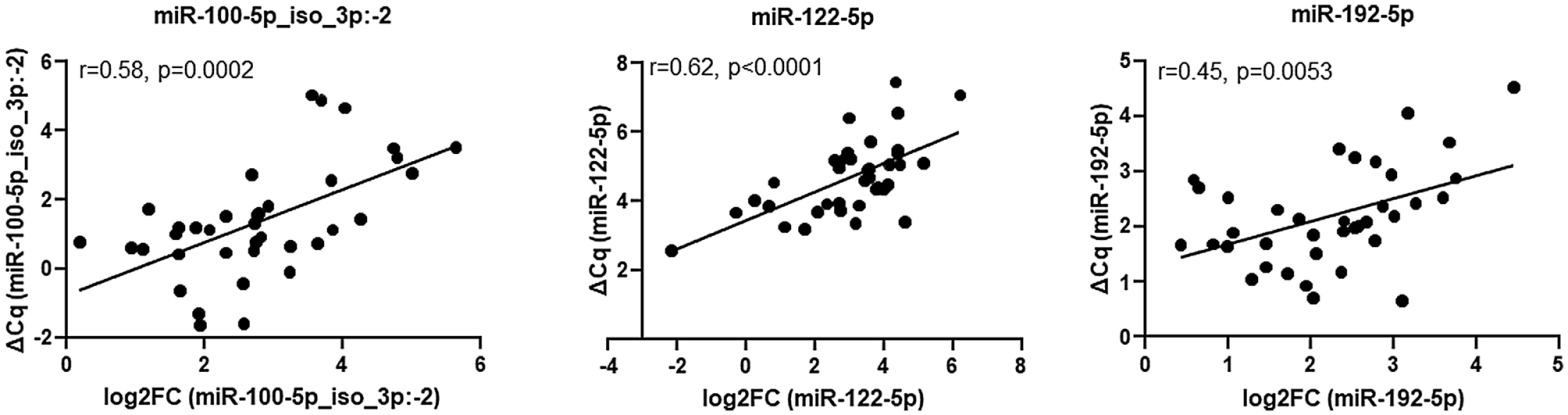
The study RT-qPCR assay was technically validated by high-throughput small RNA sequencing. To compare results from sequence analyses (log2 fold change [FC] in the base mean count) with results from qPCR analyses (ΔCq), a Pearson linear correlation was performed. The analytical variability between high-throughput RNA sequencing and the RT-qPCR–based assay to predict liver disease progression in individuals with HIV-1/HCV coinfection shows a high concordance.

**Figure 6:**
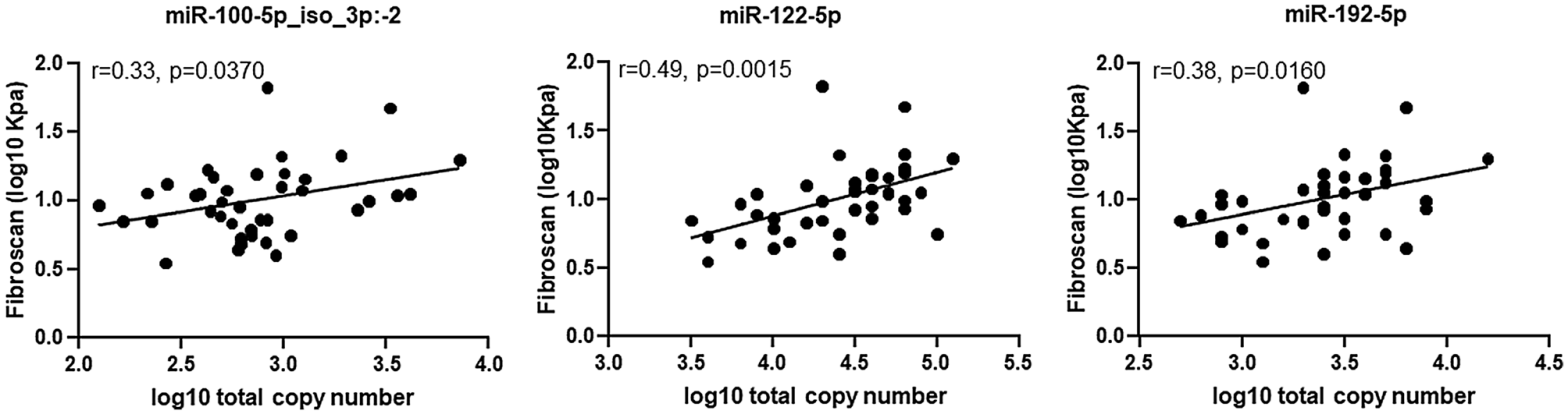
Pearson linear correlations between transient elastography liver fibrosis scores and RT-qPCR–determined plasma levels of miR-100-5p_iso_3p:−2, miR-122-5p, and miR-192-5p. Similar to the data obtained with high-throughput small RNA sequencing^15^, a significant association with liver fibrosis was also observed in this group of individuals with the data obtained using the RT-qPCR assay.

To compare the performance of the RT-qPCR assay described here, we correlated the RT-qPCR data for the miR-100-5p_iso_3p:−2 variant and the canonical miR-122-5p and miR-192-5p with the large-scale deep sequencing data on the study’s 57 plasma samples. For the three miRs we explored, there was a concordance between data from high-throughput RNA sequencing and RT-qPCR of study patients and healthy controls (miR-100-5p_iso_3p:−2: *r* = 0.55, *p* < 0.0001; miR-122-5p: *r* = 0.44, *p* = 0.0005; and miR-192-5p: *r* = 0.72, *p* < 0.0001; Fig. 4). Subsequently, we evaluated the analytical variability between high-throughput RNA sequencing and the RT-qPCR-based assay in predicting liver disease progression in individuals with HIV-1/HCV coinfection. We observed a high concordance between the two data sets (Fig. 5). Similar to the deep-sequencing data that previously indicated a significant association of miR-100-5p, miR-122-5p, and miR-192-5p plasma levels with liver fibrosis scores in individuals coinfected with HIV-1/HCV^15^, we observed a significant association of these miRs with liver fibrosis using the data from our in-house RT-qPCR assay (Fig. 6). Finally, as determined by our RT-qPCR assay, miR-100-5p_iso_3p:−2, miR-122-5p, and miR-192-5p levels were significantly upregulated in individuals coinfected with HIV-1/HCV when compared with uninfected individuals [fold change 2.7 (95% confident interval (CI) 2.2–2.9), 15.5 (95% CI 14.7–17.0) and 1.9 (95% CI 1.8–1.9), respectively) (*p *= 0.027, *p* < 0.0001, and *p* = 0.026, respectively). Overall, these results demonstrate the utility of the simple and cost effective RT-qPCR assay described here for reliable detection and quantification of circulating plasma levels of different isomiR and miR variants and to perform disease diagnostic or prognostic differential analysis.

## Discussion

Prior studies have documented the development of several RT-qPCR–based approaches to detecting and quantifying miRs. RT-qPCR is probably the most popular technique for miR detection and quantification because it is easy to use and cost-effective^6,19–24^. Commercial suppliers have developed RT-qPCR-based methods to quantify different miRs (e.g., Applied Biosystems’s Taqman MicroRNA assays), with only slight differences among them. However, current commercial RT-qPCR methods sometimes cannot discriminate isomiR variants of mature miRs with sequence endpoint differences^8,19,20,25^. Recently, both 5′ and 3′-miR length variations have been linked to different biological functions, emphasising the functional importance of isomiR variants^12,26^. In this study, we tested an in-house RT-qPCR assay to specifically detect and quantify circulating individual isomiR and miR sequences in a cohort of human plasma samples.

We found that our application of a previously described RT-qPCR method for miR quantification^18^, which uses SYBR Green detection chemistry and two microRNA-specific DNA oligonucleotides, could detect and specifically quantify miR-100-5p_iso_3p:−2, miR-122-5p, and miR-192-5p in 57 human plasma samples. Of note, miR-100-5p_iso_3p:−2 and miR-100-5p were not detected by a commercial TaqMan RT-PCR kit. Results of the RT-qPCR assay described and tested here showed good and significant correlation with the isomiR and miR quantification performed by high-throughput small RNA sequencing^15^. These findings extend previous studies^8,19,20,25^ and confirm that commercial assays or RT-qPCR targeting canonical miR sequences may not have the specificity needed for reliable isomiR quantification. In addition, the simple RT-qPCR assay described here can perform differential expression analysis and detect significant expression differences similar to those obtained with high-throughput sequencing^15^. This method should be easily adaptable to other identified isomiRs and/or miRs.

Our previous evaluation of the 57 samples studied here demonstrated that differential expression analysis of the miR-100-5p_iso_3p:−2 variant yielded better results than analysis for total miR-100-5p^15^. Compared to the total number of sequences associated with miR-100-5p, the miR-100-5p iso_3p:−2 variant sequences displayed a better correlation with liver fibrosis and better diagnostic performance. As we demonstrate here, small RNA deep sequencing analysis of miR-100-5p showed that expression of the canonical variant was very low. Consequently, isomiR variants might provide higher accuracy in differential expression analyses because of their specificity and could serve as better biomarkers for differentiating among conditions of interest^15^. Furthermore, isomiRs may be more informative than miRs as biomarkers for differentiating cancer types^14^, and their relative expression levels differ across sex and population boundaries^27^. An example is the different expression profiles of isomiRs in melanoma samples compared with the reference, canonical form^28^. It also has been established that intracellular levels of isomiRs are differentially regulated upon stimulation (e.g., by type I interferon)^19^. isomiRs can exhibit differential functions and cellular localisation according to their 3′-end length^26^. Moreover, single-cell small RNA sequencing data have revealed altered regulatory functions of different classes of isomiRs when compared with their respective canonical miRs, again supporting a biological role for many of the expressed isomiRs^13^.

miR-122-5p and miR-192-5p blood levels have been widely associated with liver injury, cirrhosis, and hepatocellular carcinoma^29,30^. The relevance of these miRs also has been assessed in individuals with HIV-1 and HIV-1/HCV^15,31–33^(Martinez, Tural and Franco submitted 2022). In contrast, until recently, the relationship between liver injury and miR-100-5p has not been as extensively described as that of miR-122-5p and miR-192-5p^15^. One possible explanation is the widespread use of commercial RT-qPCR assays to detect circulating miRs^29^. Our findings indicate the relevance of identifying and properly detecting and quantifying isomiRs with physiological or pathological significance.

Although our results are limited to the data obtained with three miR sequences and further work should be done to assess other isomiR variants, these findings demonstrate the utility of this simple, cost-effective RT-qPCR assay to detect and quantify different isomiRs and miRs. Another possible limitation of our study is the lack of an exact correlation between RT-qPCR and miR sequence data. One possible explanation may be the lack of normalization of the RT-qPCR data (i.e. raw Cq values were correlated), while the sequencing data was normalized. A better understanding of the differential expression of specific isomiRs in physiological or pathogenic situations may provide the basis for further functional studies and complement small RNA approaches in the identification of better disease biomarkers.

## Materials and methods

### Study sample cohort

All individual study plasma samples were previously described in a cross-sectional study on miR signatures that can predict liver fibrosis progression in individuals with HIV-1/HCV coinfections^15^. The 57 study samples included 19 from uninfected healthy controls, 23 from HIV-1/HCV coinfected individuals who experienced progression to liver fibrosis, and 15 from HIV-1/HCV individuals who did not experience progression to liver fibrosis. The clinical characteristics of the 57 study participants have been described previously^15^. Individual samples were obtained with written informed consent, as required for Institutional Review Board-approved protocols. This miRs study was approved by the Ethic Board of the Hospital Universitari Germans Trias I Pujol (CEI: PI-18-132). All research was performed in accordance with relevant guidelines/regulations.

### RNA extraction

Total RNA was extracted from 100 µl of plasma previously centrifuged and cleared for 5 min at 3000×*g*, with the phenol-free MagMAX mirVana Total RNA Isolation Kit (Thermo Fisher Scientific), following the manufacturer’s instructions. RNA was eluted in 50 µl of pre-heated elution buffer at 60 °C and stored at − 30 °C.

### RT-qPCR

Two microlitres of the total extracted RNA were used for the cDNA amplification with the TaqMan Advanced miRNA cDNA Synthesis Kit (Applied Biosystems/Thermo Fisher Scientific) and following the manufacturer’s instructions. We used 2 µl of the miRAmp amplification in a QuantStudio 5.0 real-time PCR System (Applied Biosystems/Thermo Fisher Scientific). Real-time PCR was performed in a total volume of 20 µl containing 2 µl of the miRAmp reaction, 10 µl of the TaqMan Fast Advanced Master Mix (2X; Applied Biosystems/Thermo Fisher Scientific), 1 µl of the 20X TaqMan Advanced microRNA Assay, and 7 µl of RNase-free water. Conditions of the real-time program were 95 °C (20 s), followed by 50 cycles (95 °C 3 s plus 60 °C 30 s). TaqMan Advanced MicroRNA Assays (Applied Biosystems/Thermo Fisher Scientific) were used for the detection of miR122-5p (477855_mir), miR192-5p (478262_mir), and miR100-5p (478224_mir). Samples were run in duplicate in a 96-well reaction plate.

For absolute miR RT-qPCR (SYBR), we used 2 µl of total RNA (previously extracted with MagMAX mirVana Total RNA Isolation Kit; Applied Biosystems/Thermo Fisher Scientific) as an input sample for the cDNA amplification, following previously published methodology^18^ with some modifications. Briefly, 2 µl of total RNA was reverse transcribed in two steps. The first step consisted of the addition of a poly A tail using 1 µl of 10X poly A buffer (New England Biolabs), 1 µl of ATP 10 mM, and 0.25 µl (1.25 U) of *E. coli* Poly A polymerase (5000 U/ml; New England Biolabs), in a final volume of 10 µl at 37 °C (30 min). In this step, we added 10^5^ miR-RNU6B copies, an internal control not found in our human plasma samples. In the second step, poly-adenylated miRs were reverse-transcribed with the addition of the total poly A tailing reaction (10 µl) into the RT reaction in a total volume of 20 µl at 37 °C (50 min) plus an inactivation step at 70 °C (15 min). The reverse transcription mix contained 4 µl of 5X MMLUV-RT buffer (Invitrogen), 0.4 µl of dNTP 25 mM, 2 µl RT-primer 10 µM, 1 µl M-MuLV-RT (Invitrogen/Thermo Fisher Scientific), 2 µl DTT 0.1 M, 0.5 µl RNasin 40 U/µl (Promega), and 0.1 µl RNAse-free water. The RT oligonucleotide was 5′-CAGGTCCAGTTTTTTTTTTTTTTTVN-3′^18^. To determine the total copy number of miRs, we added 1 µl of the obtained cDNA with 0.625 µl of each miR-specific DNA oligonucleotide (forward and reverse) at final concentrations of 250 nM, along with 12.5 µl of 2X SYBR Green PCR Master mix (Applied Biosystems/Thermo Fisher Scientific) in a total volume of 25 µl. Forward oligonucleotides for miR-100-5p, miR-100-5p_iso3p:−2, miR-122-5p, and miR-192-5p were 5′-CAGAACCCGTAGATCCGA-3′, 5′-CAGAACCCGTAGATCCGA-3′, 5′-GCAGTGGAGTGTGACAATG-3′, and 5′-CAGCTGACCTATGAATTGACA-3′, respectively. Reverse oligonucleotides were 5′-GTCCAGTTTTTTTTTTTTTTTCAAG-3′, 5′-GGTCCAGTTTTTTTTTTTTTTTCAAGT-3′, 5′-CCAGTTTTTTTTTTTTTTTCAAACACC-3′, and 5′-TCCAGTTTTTTTTTTTTTTTGGCT-3′, respectively. Control RNU6B forward and reverse oligonucleotides were 5′-ACACGCAAATTCGTGAAGCGTTCCA-3′ and 5′-CAGGTCCAGTTTTTTTTTTTTTTTAAA-3′, respectively. Samples were run in triplicate and in parallel with the specific miR and the RNU6B standard curves in a 96-well reaction plate. The absolute miR qPCR amplification was performed in the QuantStudio 5.0 Real-Time PCR system (Applied Biosystems/Thermo Fisher Scientific) with the standard protocol for the SYBR Green reactions (Applied Biosystems/Thermo Fisher Scientific). The qPCR program used was 2 min at 50 °C followed by 10 min at 95 °C, 40 cycles of 1 5 s at 95 °C plus 1 min at 60 °C, followed by a dissociation stage (15 s at 95 °C, 1 min at 60 °C, and 1 s at 95 °C). The melting curve and the corresponding result of the dissociation stage were used in each run to ensure amplification specificity. The total miR copy number was obtained with extrapolation of the Cq obtained per sample onto the standard curve and calculated automatically with QuantStudio Design and Analysis software (Applied Biosystems/Thermo Fisher Scientific). On each 96-well plate, a positive control (10^4^ copies of the cDNA derived from each miR synthetic oligonucleotide) was added to confirm the detection on the standard curve. As negative controls, we tested 10^4^ copies of the other miRs and the RNU6B to be sure that we had no cross-amplification, along with using a no-template control.

We used miRprimer software^34^ to design the miR-specific DNA primers (forward and reverse) used in the absolute miR qPCR amplification of our three miR targets, specifically for the isoform miR-100-5p_iso3p:−2. For designing each specific miR standard curve, and as a template for cDNA determination, we used 10 ng of a synthetic RNA oligonucleotide (RNase-Free HPLC purification, IDT DNA technologies) based on the sequence of each miR: miR-100-5p (5′-AACCCGUAGAUCCGAACUUGUG-3′), miR-100-5p_iso_3p:−1 (5′-AACCCGUAGAUCCGAACUUGU-3′), miR-100-5p_iso_3p:−2 (5′-AACCCGUAGAUCCGAACUUG-3′), miR-100-5p_iso_3p:−3 (5′-AACCCGUAGAUCCGAACUU-3′), miR-100-5p_iso_3p:−4 (5′-AACCCGUAGAUCCGAACU-3′), miR-122-5p (5′-UGGAGUGUGACAAUGGUGUUUG-3′), miR-192-5p (5′-CUGACCUAUGAAUUGACAGCC-3′), and RNU6B (5′-CGCAAGGAUGACACGCAAAUUCGUGAAGCGUUCCAUAUUUUU-3′). The cDNA standard curve was generated by tenfold dilutions (10^1^–10^7^) in TE 1X buffer (IDT DNA technologies) and stored at − 30 °C in small aliquots (8 µl) to avoid freeze-and-thaw cycles.

### miR sequence data

The miR sequence data are publicly available in the GEO database under accession number GSE161845 (http://www.ncbi.nlm.nih.gov/geo/).

### Statistical analyses

We used Pearson correlation to evaluate the dynamic range and sensitivity of the tested RT-qPCR, correlation of plasma miR levels between RT-qPCR and Illumina large-scale deep sequencing analysis, validation of the study RT-qPCR assay by high-throughput small RNA sequencing, and the association between miR levels and liver fibrosis (transient elastography measurements). To compare mean values between groups, we used the Mann–Whitney U test. All statistical analyses were performed with GraphPad Prism version 8.3.0 for Windows (GraphPad Software, San Diego, California, USA).

## Supplementary Information

Supplementary Figure 1.

Supplementary Figure 2.

Supplementary Table 1.

## Data Availability

All data generated or analyzed during this study are included in this manuscript (and in its Supplementary Information file).
