# Supplementary figures and images for: Rapid and accurate quantification of isomiRs by RT-qPCR
Source: Sci Rep. 2022 Oct 14;12:17220. doi: 10.1038/s41598-022-22298-7 (PMC9568571; doi:10.1038/s41598-022-22298-7)

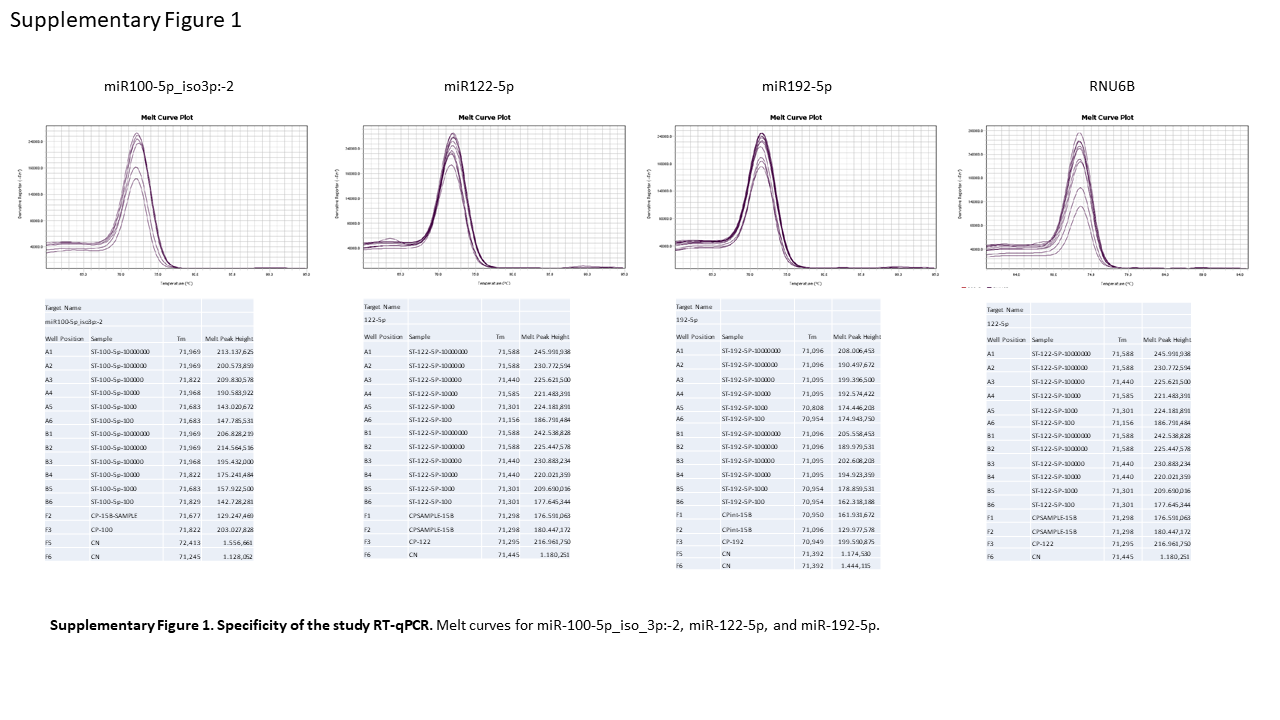

Supplement: Supplementary file 1 — Supplementary Figure 1. [file 41598_2022_22298_MOESM1_ESM.tif]

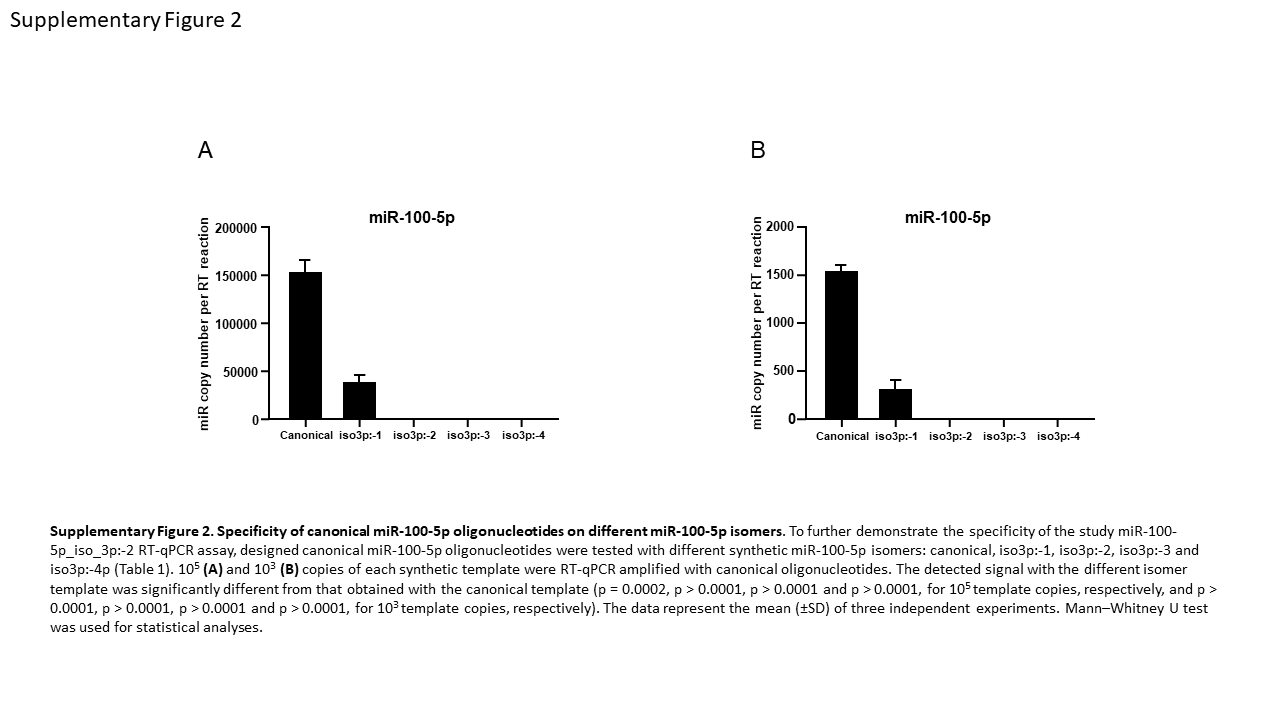

Supplement: Supplementary file 2 — Supplementary Figure 2. [file 41598_2022_22298_MOESM2_ESM.tif]
